# On the assessment of the ability of measurements, nowcasts, and forecasts to track changes

**DOI:** 10.1186/s12874-024-02397-x

**Published:** 2024-11-12

**Authors:** Jonas Rieger, Bolin Liu, Bernd Saugel, Oliver Grothe

**Affiliations:** 1 Institute for Operations Research, Karlsruhe Institute of Technology (KIT), Kaiserstr. 12, Karlsruhe, 76131 Germany; 2 Department of Anesthesiology, Center of Anesthesiology and Intensive Care Medicine, University Medical Center Hamburg-Eppendorf, Martinistrasse 52, Hamburg, 20246 Germany

## Abstract

**Background:**

Measurements, nowcasts, or forecasts ideally should correctly reflect changes in the values of interest. In this article, we focus on how to assess the ability of measurements, nowcasts, or forecasts to correctly predict the direction of changes in values - which we refer to as the ability to track changes (ATC).

**Methods:**

We review and develop visual techniques and quantitative measures to assess ATC. Extensions for noisy data and estimation uncertainty are implemented using bootstrap confidence intervals and exclusion areas.

**Results:**

We exemplarily illustrate the proposed methods to assess the ability to track changes for nowcasting during the COVID-19 pandemic, patient admissions to an emergency department, and non-invasive blood pressure measurements. The proposed methods effectively evaluate ATC across different applications.

**Conclusions:**

The developed ATC assessment methods offer a comprehensive toolkit for evaluating the ATC of measurements, nowcasts, and forecasts. These techniques provide valuable insights into model performance, complementing traditional accuracy measures and enabling more informed decision-making in various fields, including public health, healthcare management, and medical diagnostics.

## Introduction

Measurements, nowcasts, or forecasts ideally should correctly reflect changes in the values of interest. It is thus important to meticulously assess the ability of measurements, nowcasts, or forecasts to correctly predict the direction of changes in values - which we refer to as the ability to track changes (ATC). Although measurements, nowcasts, and forecasts fundamentally differ as they either measure or predict a value, similar methods can be used to assess their ATC.

Forecasting methods predict the future based on historical data, patterns, or exogenous factors. A forecast is computed based on the current value of interest and an estimate of its future development. In medicine and healthcare, forecasting - for example - is used to predict patient volumes in emergency departments [1, 2] or the demand of emergency medical services [3].

Methodologically evolved from forecasting [4], nowcasting methods focus on predictions for the present, the immediate future, and the recent past [5, 6]. Nowcasting methods use high-frequency indicators or preliminary measurements related to the value of interest and focus on updating predictions using currently available information [7]. Nowcasting, for example, can assess the current situation during an ongoing epidemic, considering the main pathogenic, epidemiological, clinical, and socio-behavioral factors [8] or provide daily numbers of COVID-19 cases for events that have occurred but have not yet been reported [9, 10].

Measurements aim to obtain accurate and precise values of a measurable quantity (measurand; [11, 12]). Repeated measurements can be used to track changes in a value over time. When introducing new measurement methods, they are evaluated against current reference methods, often called the “gold standard”, by simultaneously measuring the same quantity with the new method and the reference method - often in various individuals or different clinical settings.

In forecasting and nowcasting the evaluation of performance or goodness is usually based on statistical methods quantifying the accuracy such as the root mean square error (RMSE), probabilistic scoring rules, and calibration measures [9, 10, 13], see also Steyerberg et al. [14] for an overview in the medical context together with less known measures. However, although techniques like ROC curves for binary direction models or more general concordance measures (see, e.g., [15]) are used, there is no general measure directly framed to assess the method’s ATC. In a forecasting competition on armed conflicts, the assessment of the ATC recently gained attention as Vesco et al. [16] proposed the novel targeted absolute deviation with direction augmentation (TADDA) score with an additive tracking-changes-component for evaluation. However, the score poses an unintuitive incentive to forecasters and is thus theoretically problematic [17]. When evaluating the performance or goodness of a measurement method like in classical method comparison studies, comparative statistics such as Bland-Altman analysis [18] and the percentage error [19] are commonly used. In this strand of literature, the best way to assess the ATC of measurement methods is a field of active ongoing research [20–24].

In this article, we focus on how to assess the ability of measurements, nowcasts, or forecasts to track changes. We formalize the concept of ATC and present visual techniques and quantitative measures to assess it - considering both noiseless data and data with noise and small non-informative changes. We introduce the conditional ATC plot, a new graphical method for assessing the local ability, and review bootstrap methods for calculating confidence intervals. We extend the concept of assessment to probabilistic predictions. We exemplarily illustrate the proposed methods to assess the ATC for nowcasting during the COVID-19 pandemic, patient admissions to an emergency department, and non-invasive blood pressure measurements - and thus provide blueprints for future assessments. We discuss practical implementation and interpretation of the measures, thus providing the basis for communicating model limitations to forecasters, nowcasters, and public health officials. Ready-to-use code is available on https://github.com/jo-rie/aatc.

## Assessment of the ability to track changes (ATC)

### Computing changes and notation

We base the assessment of ATC on the measured/observed/true and the predicted changes in a value of interest over a time horizon *l*. The *true* change is straightforward to compute for all types of measurement, nowcast, or forecast. Let $\textbf{y} = (y_t)_{t=0}^T$ denote the actual values for nowcasting or forecasting, or gold standard measurements up to time *T*. The sequence of changes is then given by the differences of values in $\textbf{y}$ with horizon *l*, that is,

1 $$\begin{aligned} \textbf{y}^{\Delta ,l}_t = (y_{t} - y_{t-l}) \quad \text {for}\ t = l, \dots , T. \end{aligned}$$

The definition of the *predicted* change depends on the context; Table 1 summarizes the notation for measurements, nowcasts, or forecasts and the computation of the predicted change. While the computation for measurements is straightforward and well-established, we develop the framework for nowcasts and forecasts in the following sections. For nowcasting, let $x_{t | \tau }$ denote the nowcast for time *t* computed with the knowledge of time $\tau$. We call *t* the *target time* and $\tau$ the *issue time*. The predicted change is computed by

2 $$\begin{aligned} \textbf{x}^{\Delta ,l} = \left\{ \begin{array}{ll} (x_{t|t} - x_{t-l|t})^T_{t=l} & \text {if}\ y_{t-l}\ \text {is not known at time}\ t, \\ (x_{t|t} - y_{t-l})^T_{t=l} & \text {otherwise}. \end{array}\right. \end{aligned}$$

**Table 1:** Computation of the predicted change in the different applications. For nowcasting and forecasting, $x_{t | \tau }$ refer to values issued at $\tau$ with a target time *t*. For measurement, $x_t$ denotes the test device measurement at time *t*

Application	Predicted change computation
Measurement	$(x_{t} - x_{t-l})_{t=l}^T$
Nowcasting	$$\textbf{x}^{\Delta ,l} = \left\{ \begin{array}{ll} (x_{t|t} - x_{t-l|t})^T_{t=l}, & \text {if } y_{t-l} \text { is not known at time } t, \\ (x_{t|t} - y_{t-l})^T_{t=l}, & \text {otherwise}. \end{array}\right.$$
Forecasting	$\textbf{x}^{\Delta ,l} = (x_{t|t-l} - y_{t-l})^T_{t=l}$

When computing the predicted change of a nowcast for a time *t*, we use the best knowledge available at that time *t*, and the true value might not be known yet. If the true value $y_{t-l}$ is known at time *t*, the predicted change is computed by the difference between the nowcast and the true value, as $y_{t-l}$ is also known by the nowcaster and incorporated into the nowcast. Through the computation in Eq. (2), the predicted change can be computed with the knowledge of the nowcaster at time *t*.

The notation is similar for forecasting: Let $x_{t | \tau }$ denote the forecast for target time *t* and issue time $\tau$. The predicted change is computed by

3 $$\begin{aligned} \textbf{x}^{\Delta ,l} = (x_{t|t-l} - y_{t-l})^T_{t=l} \end{aligned}$$

with the same structure as in the nowcasting case and consistent indices with $\textbf{y}^{\Delta ,l}$. If the true value $y_{t-l}$ is not known at time $t-l$, a similar modification can be made as in Eq. (2).

The distinction between forecast and issue time is unnecessary in measurement analysis, as the measurement is typically available with a very short time lag. Thus, $x_t$ denotes the test method measurement for time *t*. The computation

4 $$\begin{aligned} (x_{t} - x_{t-l})_{t=l}^T \end{aligned}$$

yields the change by the test method. It is computed purely by the test method without the gold standard $y_t$ to analyze whether the gold standard and test method changes are consistent. Accordingly, $y_{t-l}$ is not used in the computation even if known at time *t* in contrast to forecasting and nowcasting.

In applications, data are often not available for all time steps, for example, due to technical problems or delays in data transfer (see the examples in Nowcasting during the COVID-19 pandemic and Forecasting patient admissions to an emergency department sections). We refer to time steps for which either measurement, nowcast, or forecast or true values are unavailable as missing values. Systematical missing values could lead to a biased assessment, and missing data should be inspected for any underlying patterns. If the missing values are not systematic, random, and occur scarcely, data pairs with missing values can be excluded from the data to calculate the measures (see [25], Section 1.3). Note that in the case of measurement data, one missing value in the time series leads to two undefined differences in the change series; that is if $x_t$ is missing, $x^{\Delta}_{t}$ and $x^{\Delta }_{t+l}$ are undefined; if an observation $y_t$ is missing, $y^{\Delta}_{t}$ and $y^{\Delta }_{t+l}$ are undefined. The data pair is excluded even if the corresponding nowcast or forecast is available.

### The four-quadrant plot

**Fig. 1:**
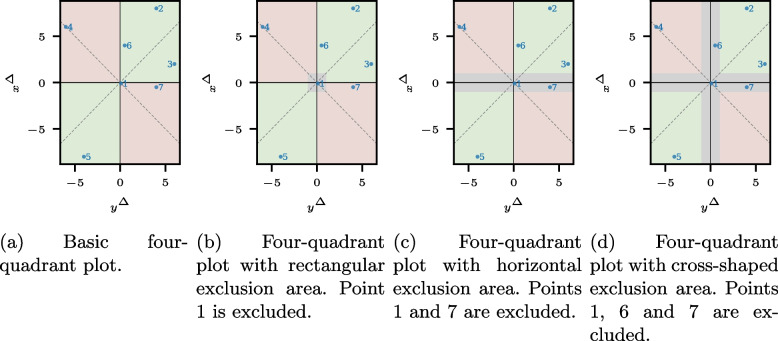
Illustrations of the four-quadrant plot with sample points and with and without exclusion areas. The rectangular exclusion area in Fig. 1**b** excludes only points where both components are likely to be noise-driven, while the exclusion areas in Fig. 1**c** and **d** exclude points where at least one component is noise-driven

**Fig. 2:**
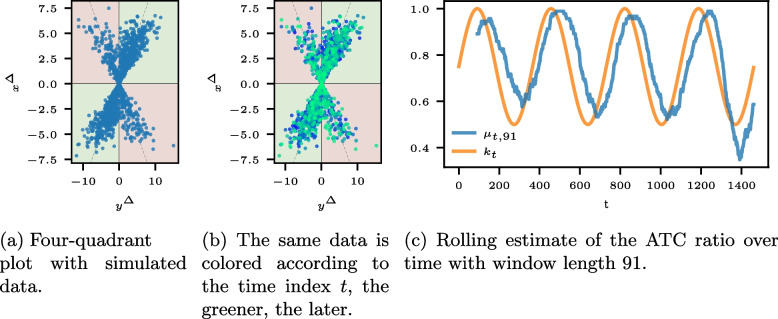
Visualizations for data with a time-varying ATC ratio. We defer information on the data generation process to the Appendix A (see Data generation for Assessment of the ability to track changes (ATC) section). The ATC ratio for the entire data set is $\mu = 0.7577$. The strong seasonality of the ATC ratio becomes visible in Fig. 2**c**. The green curve $k_t$ shows the theoretical probability that $x^{\Delta}_{t}$ has the same sign as $y^{\Delta}_{t}$ for each time step. The ATC ratio has a pronounced sinus-shaped seasonality with a peak after a quarter of a year and a low point after three quarters. The rolling estimates detect the yearly course of the ATC ration. Naturally, they are shifted to the right compared to $k_t$ as the windows look backward

Formally, the assessment of ATC is the same for measurements, nowcasts, and forecasts, given the notation for the respective application of Computing changes and notation section. In the following, we omit the horizon *l* for ease of notation; $\textbf{x}^{\Delta }$ and $\textbf{y}^{\Delta }$ refer to $\textbf{x}^{\Delta ,l}$ and $\textbf{y}^{\Delta ,l}$ for a common horizon *l*. The ATC is maximal if all predicted change directions are correct; that is, the sign of all elements of $\textbf{x}^{\Delta }$ and $\textbf{y}^{\Delta }$ coincide. Consequently, when assessing the ATC, we examine the statistical consistency of $\text {sign}(\textbf{x}^{\Delta })$ and $\text {sign}(\textbf{y}^{\Delta })$. A simple yet insightful method is the four-quadrant plot, which is well-established in measurement analysis and can be extended to nowcasts and forecasts (see, e.g., [22, 26]). In a four-quadrant plot, the occured changes and the predicted changes are plotted together, that is, $(y^{\Delta}_{t}, x^{\Delta}_{t})$ for $t = l, \dots , T$. Thus, the x-axis of a four-quadrant plot shows the true value differences, whereas the y-axis displays the prediction data differences. Points in the green upper right and lower left quadrants reflect a correct change direction for the respective time step, whereas points in the remaining red quadrants show incorrectly predicted changes. Figure 1a displays a basic four-quadrant plot, and Fig. 2a shows a four-quadrant graph for simulated data with $T=1461$, for example, four years of daily data (for the data generation, see Appendix A: Data generation for Assessment of the ability to track changes (ATC) section).

The four-quadrant plot can be extended by including information on the time index in the point color to reveal effects over time. In Fig. 2b, the point colors turn from blue to green for higher time indices *t*, that is, more recent values; $(y^{\Delta }_{l}, x^{\Delta }_{l})$ is blue and turns green until $(y^{\Delta }_{T}, x^{\Delta }_{T})$. However, four-quadrant plots become crowded for larger datasets, and sequential information on the differences is complex to assess thoroughly.

The four-quadrant plot is intuitive to interpret, and the magnitude and direction of change are shown simultaneously. Other visualization techniques, such as polar plots, lack the four-quadrant plot’s clarity and intuition without adding more information on the ATC [22].

### The ATC ratio and other measures

Analyzing the number of points in the green versus red quadrants is a standard approach in the ATC assessment of measurement data [20, 22], which we extend here to forecasts and nowcasts. With that, we estimate the probability of a correctly predicted change direction, $P(X^{\Delta } Y^{\Delta }> 0)$, where $Y^{\Delta }$ and $X^{\Delta }$ denote random variables for future incremental changes. Since $z_1 z_2> 0$ imposes the same condition as $\text {sign}(z_1) = \text {sign}(z_2)$ ($z_1, z_2 \in \mathbb {R} \setminus \{ 0 \}$), the standard estimator for $P(X^{\Delta } Y^{\Delta }> 0)$ is

5 $$\begin{aligned} \mu (\textbf{x}^{\Delta }, \textbf{y}^{\Delta }) {:=} \frac{\sum _{t \in \mathcal {T}} \mathbbm{1}\{x^{\Delta}_{t} y^{\Delta}_{t}> 0\}}{|{T}|}. \end{aligned}$$

Here, the numerator counts the number of same-sign-changes, while the denominator is the number of considered pairs $(y^{\Delta}_{t}, x^{\Delta}_{t})$. Thus, $\mu$ is the proportion of concordant changes on all changes. We refer to this estimator as the *ATC ratio* of the prediction and set $\mathcal {T} = \{l, \dots , T\}$. Visually, the measure computes the fraction of points in the upper right or lower left quadrant. Similar evaluations are used in other scientific areas, for example, with contingency tables in dichotomous forecasting or with confusion matrices in classification analysis (see, e.g., the introductions in [27], Ch. 4, and [28], Ch. 3). Many other measures can be adapted from those fields to deepen the analysis. Two simple measures that focus on a positive or negative predicted change are the positive and negative ATC ratios $\mu ^{+}$ and $\mu ^{-}$, respectively. They are defined as

6 $$\begin{aligned} \mu ^{+} (\textbf{x}^{\Delta }, \textbf{y}^{\Delta }) & {:=} \frac{\sum _{t \in \mathcal {T}} \mathbbm{1}\{x^{\Delta}_{t} y^{\Delta}_{t}> 0\} \mathbbm{1}\{x^{\Delta}_{t}> 0\}}{\sum _{t \in \mathcal {T}} \mathbbm{1}\{x^{\Delta}_{t}> 0\}},\ \text {and} \end{aligned}$$

7 $$\begin{aligned} \mu ^{-} (\textbf{x}^{\Delta }, \textbf{y}^{\Delta }) & {:=} \frac{\sum _{t \in \mathcal {T}} \mathbbm{1}\{x^{\Delta}_{t} y^{\Delta}_{t}> 0\} \mathbbm{1}\{x^{\Delta}_{t} < 0\}}{\sum _{t \in \mathcal {T}} \mathbbm{1}\{x^{\Delta}_{t} < 0\}}. \end{aligned}$$

They estimate the probability of a correct prediction of the direction of change, given that the predicted direction is positive or negative, that is, $P(X^{\Delta } Y^{\Delta }> 0 | X^{\Delta }> 0)$ and $P(X^{\Delta } Y^{\Delta }> 0 | X^{\Delta } < 0)$.

Rolling estimates of the above measures detect changes in performance over time and can give a sharper estimate of the current ATC. For the ATC ratio, a rolling estimate with a backward-looking window of length *w* at time *t* is given by

$$\begin{aligned} \mu _{t; w} (\textbf{x}^{\Delta }, \textbf{y}^{\Delta }) {:=} \frac{\sum _{t^\star = t-w + 1}^{t} \mathbbm{1}\{x^{\Delta }_{t^{\star }} y^{\Delta }_{t^{\star }}> 0\}}{w}. \end{aligned}$$

Backward-looking windows estimate the ATC ratio at a time *t* considering the *w* time steps before time *t*. The window length *w* controls the smoothing of the estimate; a larger *w* gives smoother results, while a small *w* focuses on local variations. Plotting the rolling estimates for $t = w-1, \dots , T$ yields an estimate of the ATC ratio over time. Figure 2c depicts a rolling window estimate of the ATC ratio for the simulated data of Fig. 2a and b. While colored four-quadrant plots, as in Fig. 2b, illustrate ongoing overall drifts in the ATC, seasonal aspects are only revealed in rolling window estimates.

### Accounting for noise and non-informative small changes and bootstrapping confidence intervals

The above measures can be extended to account for information on the point’s location within the quadrant. For example, points close to the zero point may have less explanatory power or may be less reliable than points far away from zero on one of the diagonals. Suppose noise or non-systematic effects are present in the true values or predictions. In that case, noise can drive a point’s assignment to a quadrant instead of a systematic ATC. This is more likely for points with at least one small coordinate.

Using an exclusion area around the zero point, as further defined below, is a straightforward and highly interpretable extension of the measures of The ATC ratio and other measures section accounting for such effects (see, e.g., [20, 22]). Points within that area are omitted in the calculation of the measures. In particular, the measurement, nowcast, or forecast is likely to have a noise component; thus, $\textbf{x}^{\Delta }$ should be subject to an exclusion area. The measures of Eqs. (5), (6) and (7) without points in the exclusion area *E* are

8 $$\begin{aligned} \mu _{e} (\textbf{x}^{\Delta }, \textbf{y}^{\Delta }, E) & {:=} \frac{\sum _{t \in \mathcal {T}} \mathbbm{1}\{\textbf{x}^{\Delta } \textbf{y}^{\Delta }> 0\} \mathbbm{1}\{(y^{\Delta}_{t}, x^{\Delta}_{t}) \notin E\}}{\sum _{t \in \mathcal {T}} \mathbbm{1}\{(y^{\Delta}_{t}, x^{\Delta}_{t}) \notin E\}},\end{aligned}$$

9 $$\begin{aligned} \mu _{e}^{+} (\textbf{x}^{\Delta }, \textbf{y}^{\Delta }, E) & {:=} \frac{\sum _{t \in \mathcal {T}} \mathbbm{1}\{x^{\Delta}_{t} y^{\Delta}_{t}> 0\} \mathbbm{1}\{x^{\Delta}_{t}> 0, (y^{\Delta}_{t}, x^{\Delta}_{t}) \notin E\}}{\sum _{t \in \mathcal {T}} \mathbbm{1}\{x^{\Delta}_{t}> 0, (y^{\Delta}_{t}, x^{\Delta}_{t}) \notin E\}}, \ \text {and} \end{aligned}$$

10 $$\begin{aligned} \mu _{e}^{-} (\textbf{x}^{\Delta }, \textbf{y}^{\Delta }, E) & {:=} \frac{\sum _{t \in \mathcal {T}} \mathbbm{1}\{x^{\Delta}_{t} y^{\Delta}_{t}> 0\} \mathbbm{1}\{x^{\Delta}_{t} < 0, (y^{\Delta}_{t}, x^{\Delta}_{t}) \notin E\}}{\sum _{t \in \mathcal {T}} \mathbbm{1}\{x^{\Delta}_{t} < 0, (y^{\Delta}_{t}, x^{\Delta}_{t}) \notin E\}}. \end{aligned}$$

The measures are then estimators for the probability of predicting the correct direction, given that the point’s location is not driven by noise or non-informative changes.

The estimators accept various shapes of the exclusion area (see Fig. 1). A rectangular exclusion area, $E = \{(y, x) \in \mathbb {R}^2: (-\varepsilon _x \leqslant x \leqslant \varepsilon _x) \wedge (-\varepsilon _y \leqslant y \leqslant \varepsilon _y) \}$ for $\varepsilon _x, \varepsilon _y> 0$, leaves out points that are small in both components. An exclusion area along one axis, for example, $E = \{(y, x) \in \mathbb {R}^2: (-\varepsilon _x \leqslant x \leqslant \varepsilon _x)\}$ for $\varepsilon _x> 0$, removes points in which one of the components could change sign by a small amount of noise. A cross-shaped exclusion area, $E = \{(y, x) \in \mathbb {R}^2: (-\varepsilon _x \leqslant x \leqslant \varepsilon _x) \vee (-\varepsilon _y \leqslant y \leqslant \varepsilon _y) \}$ for $\varepsilon _x, \varepsilon _y> 0$, along both axes accounts for the sign reversal in both components.

In most applications, the shape and size of the exclusion area can be chosen based on domain knowledge or expert opinions. The size determination can also be based on a proportion of the total variance or the total range of the data; for example, the 10% smallest absolute values in each component determine the exclusion area size. A third approach is to visualize the ATC ratio for different sizes of *E* and thus inspect the effects of the exclusion area size on the estimates. For examples of such plots, see Nowcasting during the COVID-19 pandemic section.

Confidence intervals can account for the estimation uncertainty of the measures above, an approach not yet applied in the literature. Bootstrap confidence intervals are based on resampling and not on parametric assumptions as classical confidence intervals are (for introductions see [29, 30]). Many new samples are drawn with replacement from the dataset, and the statistic of interest is computed for each sample, yielding an estimate for the distribution of the statistic of interest. Based on the derived “new” samples of the statistic, the confidence intervals can be derived through different bootstrapping methods. We use the bias-corrected and accelerated (BCa) approach for bootstrapping in the following, as it holds the confidence level for small and large samples and has a moderate computation time (see the simulation study in Appendix A: Simulation study on bootstrapping confidence intervals).

### The conditional ATC plot

The estimators described above provide information on the probabilities $P(X^{\Delta } Y^{\Delta }> 0 | X^{\Delta } Y^{\Delta } \notin E)$, $P(X^{\Delta } Y^{\Delta }> 0 | X^{\Delta }> 0, X^{\Delta } Y^{\Delta } \notin E)$, and $P(X^{\Delta } Y^{\Delta }> 0 | X^{\Delta } < 0, X^{\Delta } Y^{\Delta } \notin E)$. Notably, the visual analysis of these probabilities has not yet been addressed in the literature. A still finer analysis might be gained by considering the conditional distribution $P(X^{\Delta } Y^{\Delta }> 0 | X^{\Delta } = \chi )$ to assess the ATC of a prediction for a specific change $X^{\Delta } = \chi$ of the measurement, nowcast, or forecast. Thereby, $P(X^{\Delta } Y^{\Delta }> 0 | X^{\Delta } = \chi )$ denotes the probability of a correct direction given a predicted change of $\chi$. Thus, if a change of $\chi$ is observed in practice, one can directly assess its credibility regarding the direction. Multivariate kernel density estimation (KDE) facilitates the continuous estimation of $P(X^{\Delta } Y^{\Delta }> 0 | X^{\Delta } = \chi )$ by estimating the components $f_{X^{\Delta }, Y^{\Delta }}$ and $f_{X^{\Delta }}$ of

$$\begin{aligned} P(X^{\Delta } Y^{\Delta }> 0 | X^{\Delta } = \chi ) = \left\{ \begin{array}{ll} \int _{-\infty }^0 \frac{f_{X^{\Delta }, Y^{\Delta }}(\chi , y)}{f_{X^{\Delta }}(\chi )} \ \text {d} \, y & \text {if } \chi < 0, \\ \int _{0}^{\infty } \frac{f_{X^{\Delta }, Y^{\Delta }}(\chi , y)}{f_{X^{\Delta }}(\chi )} \ \text {d} \, y & \text {if } \chi> 0, \\ \end{array}\right. \end{aligned}$$

for $\chi \ne 0$ through a KDE. Gramacki [31] provides a comprehensive introduction to multivariate KDE, and implementations are available in many programming languages, for example, in the |statsmodels| in Python [32]. The KDE yields estimates for $P(X^{\Delta } Y^{\Delta }> 0 | X^{\Delta } = \chi )$ for all values of $\chi \in \mathbb {R}$. Multivariate KDE takes a kernel and bandwidth selector as modeling parameters. We advise using a Gaussian kernel and the cross-validation maximum likelihood as bandwidth selector (see Appendix A: Visualization of different bandwidth selectors in multivariate KDE).

Assessing $P(X^{\Delta } Y^{\Delta }> 0 | X^{\Delta } = \chi )$ graphically by drawing $P(X^{\Delta } Y^{\Delta }> 0 | X^{\Delta } = \chi )$ against $\chi$ eases the simultaneous evaluation of various $\chi$. Furthermore, the graph facilitates the comparison of various methods in a single graph, and asymmetries of $P(X^{\Delta } Y^{\Delta }> 0 | \textbf{x}^{\Delta } = \chi )$ with respect to $\chi$ in the ATC can be detected. We refer to the plot as a *conditional ATC plot*.

### Probabilistic evaluation

In nowcasting and forecasting, probabilistic predictions have become more prevalent in recent years (see Nowcasting during the COVID-19 pandemic and Forecasting patient admissions to an emergency department sections). In this section, we develop ATC assessments for probabilistic measurements and nowcasts, an approach not yet explored in the literature. Probabilistic predictions issue a probability distribution for the quantity of interest based on their available information and, thus, include a point estimate and information on the prediction uncertainty and quantiles simultaneously. Probabilistic predictions thus also contain a probability of a positive or negative change. For ATC assessment, we compare the predicted probability of positive change, denoted by $p_t$, with the occurrence of positive changes.

Probabilistic predictions can be a cumulative distribution function (CDF), probability density function (PDF), or quantiles. The CDF is the most general and can be used to derive the others, given that they exist. Let us first assume that the prediction is a CDF, and that $y_{t-l}$ is known at time *t* (see Table 1). Appendix A: Probabilistic ATC evaluation extends the analysis to quantile predictions or unknown true values.

Let for a forecast $F_{t | t-l} (x)$ denote the predicted CDF for target time *t* and issue time $t - l$, where the index is analogous to the point notation of Computing changes and notation section. The CDF $F_{t | t-l} (x)$ specifies the forecasted probability that the quantity of interest is at most *x*. A positive change occurs for any value at *t* larger than the true value $y_{t-l}$ and the CDF $F_{t | t-l} (y_{t-l})$ yields the predicted probability of any value at most $y_{t-l}$, and, thus, a negative change. Accordingly, the forecasted probability of a positive change is

$$\begin{aligned} p_t = 1 - F_{t | t-l} (y_{t-l})\quad t = l, \dots , T. \end{aligned}$$

The computation differs slightly for nowcasts, that is,

$$\begin{aligned} p_t = 1 - F_{t | t} (y_{t-l})\quad t = l, \dots , T, \end{aligned}$$

with analogous derivations as above. Let $z_t$ denote the indicator that the observed change at time *t* is positive, that is,

$$\begin{aligned} z_t = \mathbbm{1}\{y^{\Delta}_{t}> 0\} \quad t = l, \dots , T. \end{aligned}$$

The predictive power of $\textbf{p} = (p_t)_{t=l}^{T}$ for $\textbf{z} = (z_t)_{t=l}^{T}$ can be assessed using probabilistic dichotomous forecast evaluation methods. Dichotomous forecasts predict a binary outcome, such as a positive or negative change, and are evaluated numerically using scoring rules or visually through reliability diagrams.

The Brier score (BS) is a widely used scoring rule for dichotomous probabilistic forecasts [33]. In our context, it is

$$\begin{aligned} BS (\textbf{p}, \textbf{z}) = \frac{1}{T-l+1} \sum _{t=l}^{T} (p_t - z_t)^2. \end{aligned}$$

Lower values indicate the considered method’s higher probabilistic ATC. The BS assesses the calibration and sharpness of the forecast and the observation simultaneously [34, 35]. Calibration refers to the statistical consistency of forecasts and observations; that is, the event occurs with the issued probability and is considered the more fundamental quality [13]. Sharpness refers to the spread of the forecast; probabilities close to zero and one are preferable as they convey a higher certainty.

Graphical methods are a standard tool for evaluating the calibration of probabilistic forecasts in detail. In dichotomous forecasting, the reliability diagram is frequently used [34]. The reliability diagram plots the observed frequency of the positive outcome against the (binned) predicted probability. For example, it shows the proportion of observed increases, given that the predicted probability of increase was approximately 0.7. Ideally, the predicted probability equals the observed frequency, and the reliability diagram is a 45-degree line. Local deviations from the 45-degree line indicate a miscalibration for specific forecast probabilities. Thus, the reliability visualizes the local and overall calibration simultaneously. For an example of a reliability diagram, see Forecasting patient admissions to an emergency department section.

## Application to medical/healthcare nowcasting, forecasting, and measurement data

### Nowcasting during the COVID-19 pandemic

In Germany, the seven-day hospitalization rate was established as a central steering measure in November 2021 during the COVID-19 pandemic, and the imposition of severe public restrictions was based on it [36]. However, the publication of the definite hospitalization rate was substantially delayed and partially flawed for two main reasons. First, the reporting process was delayed because - among other reasons - different authorities were involved in passing the data to the RKI [37]. Second, the seven-day hospitalization rate allocated all COVID-19-related hospitalizations to the date of the first positive test (for a detailed description, see [10]). The COVID-19-Nowcasting-Hub [38] collected various nowcasts in a predefined setup, including the mean, median and other quantiles of the predicted seven-day hospitalization rate (for further information see [10] and Table 9 in Appendix B for the abbreviations used). In addition to those nowcasts, Wolffram et al. [10] construct two ensemble methods using the ensembles’ mean or median. We denote them by ENS-MEAN and ENS-MED. In line with the initial study design, we consider the period from November 22, 2021, to April 29, 2022, as the evaluation period. We use the data from February 8, 2024, for the true values and focus on nowcasts for all inhabitants of Germany. Figure 12 in Appendix B displays the true and nowcast data for the evaluation period. The time comprises the fourth wave’s end in December 2021 and nearly the entire fifth wave of the pandemic in Germany, lasting until May 28, 2022 [39].

Traditional methods often focus on point or distributional accuracy of hospitalization rates, but understanding the direction and reliability of changes is crucial for effective decision-making. The ATC assessment offers easy-to-interpret insights and helps determine not only if rates are rising or falling, but also how confidently we can make this determination. For instance, if hospitalization rates are rising, public health measures may need to be tightened. Conversely, falling rates might justify loosening restrictions. The ATC assessment’s ability to reveal asymmetries is especially valuable, as it can show whether certain models are more adept at recognizing decreases than increases, or vice versa. This information, which is not readily apparent from traditional methods, can significantly impact the interpretation of nowcasts and subsequent policy decisions for public health officials.

#### Results

Table 2 summarizes the non-ATC-aware point evaluation measures for the issued mean of the different models. The best-performing models in terms of RMSE and MAE are the ILM and RKI models. The ensemble methods ENS-MED and ENS-MEAN perform worse than the best models regarding the mean location. The performance of the models is diverse, with more than twice as high RMSE values for the worst models compared to the best models.

**Table 2:** Point evaluation measures for the issued mean of the different models in COVID-19 nowcasting

Model	RMSE	MAE	Count
ILM	648	504	153
RKI	810	670	156
RIVM	820	674	159
ENS-MED	832	675	158
ENS-MEAN	841	666	158
LMU	979	810	159
SZ	1,048	834	159
SU	1,127	899	159
KIT	1,161	912	159
EPI	1,513	1,006	159

“RMSE” and “MAE” are accuracy measures, while “Count” lists the number of non-missing values. The RMSE orders the models. The evaluation period comprises 159 days, and only a few nowcasts are missing (for explanations of the missing values, see [10], Tables A2, A3, and A4). Note that the high values for the EPI model could be driven by an exceptionally far-off value at the end of the evaluation period (see Fig. 12 in Appendix B)

In the following, we apply the ATC assessment for the short-term horizons one and medium-term horizons seven and 14 days. The horizons seven and 14 reflect a typical period until new policy changes are taken. We start by providing background information on the marginal distributions of the actual value and nowcast changes for the different horizons in Table 10 in Appendix B such as standard deviation and quantiles of the nowcasts and true values. The variability and general level of changes grow with the horizon: The standard deviation increases from roughly 300 for horizon one to 1,200 for horizon seven and 2,000 for horizon 14 days. Similarly, the 10%-quantile of changes, the basis for the exclusion area size, increases. The exclusion area is rectangular; a point falls within it if both $\textbf{y}^{\Delta }$ and $\textbf{x}^{\Delta }$ are below the respective 10%-quantile of the absolute changes. Thus, points are still included in the ATC assessment if they are large in one dimension but not in the other, thus ensuring that substantial changes in, for example, $\textbf{y}^{\Delta }$ are to be recognized by the nowcast and vice versa.

Table 3 lists the ATC ratios for all models without and with exclusion areas for the horizon of seven days. The ATC ratios without exclusion area range from 0.72 to 0.85 for the horizon of seven days. The negative ATC ratios are higher than the positive ATC ratios for all models. The confidence intervals for the positive and negative ATC ratios do not overlap for all models, indicating that the ATC ratios are indeed different. The 10%-quantile exclusion areas have, at most, an influence of 0.03 on the ratios. The model with the highest ATC ratio is the ILM model, and the model with the lowest is the RKI model. The confidence intervals between all models without and with exclusion areas overlap. The positive ATC ratio implies a similar ranking of the models than the overall ATC, while the negative ratio provides a different ranking, for example, for the RKI model. For the horizons of one and 14 days, we refer to Table 11 in Appendix B.

**Table 3:** The ATC ratio $\mu ^{7}$, positive ATC ratio $\mu ^{+,7}$, and negative ATC ratio $\mu ^{-,7}$ for the models without and with exclusion areas for the horizon seven days in COVID-19 nowcasting. The exclusion areas are rectangles centered on the zero points with a width and height of twice the 10%-quantile of the absolute values of nowcast and true values. The subscript $q_{0.1}$ denotes the measures with exclusion area. There are 66 positive and 93 negative actual changes in the considered time period

	$\varvec{\mu }^{\varvec{7}}$	$\varvec{\mu }^{\varvec{+,7}}$	$\varvec{\mu }^{\varvec{-,7}}$	$\varvec{\mu }^{\varvec{7}}_{\varvec{q}_{\varvec{0.1}}}$	$\varvec{\mu }^{\varvec{+, 7}}_{\varvec{q}_{\varvec{0.1}}}$	$\varvec{\mu }^{\varvec{-, 7}}_{\varvec{q}_{\varvec{0.1}}}$
EPI	0.77 (0.71, 0.82)	0.67 (0.58, 0.75)	0.87 (0.79, 0.92)	0.78 (0.72, 0.83)	0.68 (0.59, 0.77)	0.88 (0.81, 0.93)
ILM	0.85 (0.80, 0.89)	0.73 (0.64, 0.80)	0.99 (0.94, 1.00)	0.85 (0.80, 0.90)	0.74 (0.65, 0.81)	0.99 (0.94, 1.00)
KIT	0.74 (0.69, 0.79)	0.64 (0.55, 0.72)	0.87 (0.80, 0.93)	0.75 (0.69, 0.80)	0.64 (0.55, 0.72)	0.88 (0.81, 0.94)
LMU	0.80 (0.74, 0.85)	0.70 (0.62, 0.79)	0.91 (0.84, 0.95)	0.81 (0.75, 0.86)	0.72 (0.63, 0.79)	0.92 (0.85, 0.96)
ENS-MEAN	0.82 (0.76, 0.86)	0.71 (0.63, 0.79)	0.94 (0.89, 0.99)	0.82 (0.76, 0.87)	0.71 (0.63, 0.78)	0.96 (0.90, 0.99)
ENS-MED	0.82 (0.76, 0.87)	0.70 (0.62, 0.78)	0.96 (0.90, 0.99)	0.83 (0.77, 0.87)	0.72 (0.63, 0.79)	0.96 (0.90, 0.99)
RIVM	0.83 (0.77, 0.87)	0.74 (0.65, 0.81)	0.92 (0.86, 0.96)	0.83 (0.78, 0.88)	0.74 (0.65, 0.81)	0.93 (0.87, 0.97)
RKI	0.72 (0.65, 0.77)	0.60 (0.51, 0.67)	0.98 (0.92, 1.00)	0.73 (0.67, 0.78)	0.61 (0.52, 0.68)	0.98 (0.92, 1.00)
SU	0.81 (0.75, 0.86)	0.71 (0.62, 0.78)	0.92 (0.85, 0.96)	0.81 (0.75, 0.85)	0.71 (0.63, 0.79)	0.92 (0.85, 0.96)
SZ	0.78 (0.72, 0.83)	0.67 (0.58, 0.75)	0.91 (0.84, 0.96)	0.78 (0.72, 0.83)	0.67 (0.58, 0.75)	0.92 (0.85, 0.97)

**Fig. 3:**
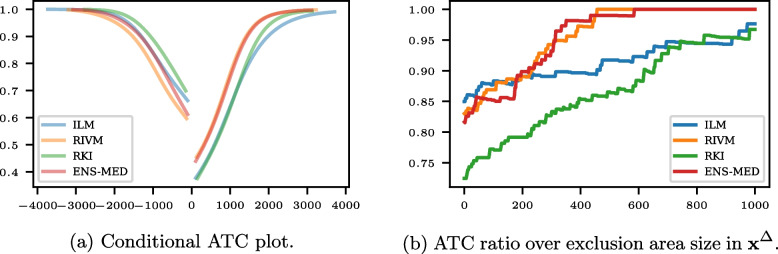
Conditional ATC plot and ATC ratio over exclusion area for the nowcasts of the seven-day hospitalization rate ILM, RKI, RIVM, and ENS-MED for the horizon seven days in COVID-19 nowcasting

Figure 3 shows the conditional ATC plots and the ATC ratio over the exclusion area for the horizon seven days; the respective plots for the horizons one day and 14 days are shown in Fig. 14 in Appendix B. Here, only the best models in point evaluation measures, ILM, RKI, RIVM, and ENS-MED, are shown to keep the plots easily readable. If RKI or ILM issues a fall in the hospitalization rate, the probability of a fall is higher than if RIVM or ENS-MED issues a fall. The opposite is the case for a nowcasted hospitalization rate increase, and the difference between the models’ performance is more prominent than for a fall. Similar observations can be made for the horizon of 14 days in Fig. 14b in Appendix B. For a horizon of one day, the models’ conditional ATC difference is less pronounced (see Fig. 14a in Appendix B). The RKI model is still less conclusive when issuing an increase in the hospitalization rate, while RIVM is most informative in that case. The curves cross for an issued fall, with ENS-MED being on top for issued falls above 250.

The ATC ratios for various exclusion areas are shown in Fig. 3b. The ATC ratio generally increases with larger exclusion areas. While the RIVM and ENS-MED ATC ratios evolve similarly, the RKI and ILM ATC ratios get closer. For the horizon of one day, the RKI ATC ratio decreases with increasing exclusion area size while the other models rise (see Fig. 14c in Appendix B). For the horizon of 14 days, all ATC ratio curves increase with the exclusion area size (see Fig. 14d in Appendix B).

**Fig. 4:**
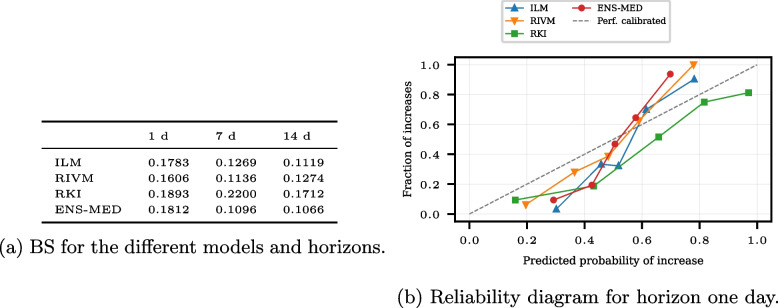
Brier scores and reliability diagrams for the COVID-19 nowcasting models ILM, RKI, RIVM, and ENS-MED. The reliability diagram bins are chosen according to the empirical quantiles of the predicted probabilities. In the computation of BS and reliability diagram, missing values are excluded. The reliability diagram for the horizons seven and 14 days is in the appendix (see Fig. 15 in Appendix B)

Figure 4 shows the Brier score (BS) and reliability diagrams for the same subset of models, the ILM, RKI, RIVM, and ENS-MED. The probabilities of increase for the different models are computed using the nowcast quantiles. For each horizon *l*, 10, 000 samples of the forecast date *t* and the forecast date $t-l$ based on the nowcasts of issue date *t* are generated, and the proportion of positive changes is computed (see Appendix A: Probabilistic ATC evaluation). Remember that a low BS and a reliability diagram along the diagonal are signs of a high ATC. The BS is the lowest for the RIVM model for a one-day-horizon, while the ENS-MED model has the best BS for the horizon of seven and 14 days. The RKI model yields the highest BS for all horizons. Note that the BS is 0.25 for random guessing; thus, all models perform better than random guessing. The reliability diagrams show that the models are not well calibrated for the horizon of one day. While for predicted probabilities below 0.5, the observed ratio of increases is smaller, it is higher than predicted for probabilities above 0.5. Figure 15c in Appendix B shows the observed predicted increase probabilities for all horizons. For the horizons of seven and 14 days, the nowcasters issue only a few moderate probabilities, and most probabilities are near zero and one.

#### Discussion

For all horizons, the influence of the exclusion area on the 10%-quantile level is negligible. For example, the ATC ratio changes at most by 0.03 for the EPI model with $\mu ^{-,14}$. The exclusion areas are thus not crucial for the ATC assessment in the case of the nowcasts of the seven-day hospitalization rate. The lower bound of confidence intervals is at least 0.68 for all models, indicating that they perform better than random guessing the trend.

ATC assessment evaluates the models differently from point evaluation measures. RKI is among the best in point evaluation measures but performs worse in ATC assessment. The assessment of asymmetry in the conditional ATC plots is crucial for interpreting the ATC ratios, with the RKI model being the most prominent example.

Figure 3b shows that larger exclusion areas increase the ATC ratio, indicating that the predicted direction is more accurate for large predicted changes.

The probabilistic ATC assessment shows that the models are better than random guessing. The reliability diagram cannot provide information if specific probabilities are issued scarcely. Thus, the reliability diagrams for the horizons of seven and 14 days do not contain information on moderate probabilities. The BS values, however, work well for those examples and provide a good measure for the ATC of the models.

A more extensive data size would be beneficial for assessing the models’ performance. For the evaluation period of 159 days, the ATC ratio confidence intervals overlap; thus, no conclusions can be drawn from the ATC assessment comparing the models.

### Forecasting patient admissions to an emergency department

In a second example, we consider forecasting patient admissions to an emergency department per hour with data and models by Rostami-Tabar, Browell, and Svetunkov [2]. Every 12 hours, the models issue hourly forecasts for the next 48 hours.

Rostami-Tabar, Browell, and Svetunkov [2] publish means and probabilistic quantile forecasts for various models and input data. We use the published mean as a point forecast for the ATC assessment and evaluate the probabilistic ATC based on the quantile forecasts subsequently. Considering only the forecasts of at least 36 hours ahead, we restrict the evaluation period to March 2, 2018, at noon, to February 28, 2019, at 23:00, comprising 8,724 hours.

While traditional methods provide point estimates, ATC assessment offers a simple, intuitive way to evaluate model performance and directional accuracy. This approach is particularly valuable for management, as it facilitates easy comparisons between expected workload and recent shifts. For instance, if staff was near capacity during the last shift, ATC assessment can clearly indicate whether an increase in patient admissions is likely, allowing management to proactively adjust resources. This directional insight, often hidden in conventional methods, enables more informed and timely decision-making.

The number of patient admissions has a strong weekly and daily pattern. Thus, we consider the horizons of 72 hours, the last already observed shift of the same hour of day, and seven days, the previous shift of the same hour and day, in ATC assessment.

#### Results

Table 4 lists the point evaluation measures and the count of available forecasts. The best-performing models regarding RMSE and MAE are the NBI-2 and Poisson-2 models. More than 8,600 forecasts are available for all models, with changes in the number due to missing values on four afternoons in 2018.

**Table 4:** Point evaluation measures for the forecasting models for patient admissions to an emergency department

Model	RMSE	MAE	Count
NBI-2	8.883	3.200	8688
Poisson-2	8.884	3.200	8688
Poisson-1	9.164	3.238	8688
Benchmark-2	9.246	3.236	8688
Ttr-2	9.394	3.266	8688
NOtr-1	9.413	3.276	8688
NOtr-2	9.413	3.276	8688
Poisson-2-I	9.458	3.276	8688
Benchmark-1	10.065	3.331	8688
GBM-2	11.663	3.542	8688
tbats	12.905	3.912	8724
Prophet	13.078	3.877	8724
qreg-1	13.337	3.758	8688
Regression-Poisson	21.162	4.818	8724
ADAM-iETSX	28.000	5.561	8724
ETS	29.358	5.742	8724

The smaller count for some models stems from missing forecasts scattered throughout the evaluation period. Note that the reported values for the RMSE differ from those in Rostami-Tabar, Browell, and Svetunkov [2] due to differences in the evaluation period

We start by analyzing the marginal distributions for the predicted and observed changes for the three- and seven-day horizons in Table 5, again. The computed difference aligns with Computing changes and notation section, that is, the difference between the forecasted mean and true value of three and seven days before, as the actual value is available when issuing the forecast. The positive change fraction varies between 0.39 and 0.63 for the horizon of three days and between 0.37 and 0.63 for the horizon of seven days. The variability of changes decreases for the larger horizon for most models; only for the ETS model does it increase. The 10%-quantile of the changes is between zero and one for all models and horizons. Thus, we use an exclusion area of size 1. The resulting fraction of included values in the computation is also listed in Table 5 and is at least 79% of the values.

**Table 5:** Marginal analysis of the forecast and true changes in patient admissions to an emergency department

	(1), l=3	$\varvec{\sigma }_{\varvec{x}^{\varvec{\Delta , 3}}}$	$\varvec{q}_{\varvec{0.1}} \varvec{(x}^{\varvec{\Delta , 3}}\varvec{)}$	(2), l=3	(1), l=7	$\varvec{\sigma }_{\varvec{x}^{\varvec{\Delta , 7}}}$	$\varvec{q}_{\varvec{0.1}} \varvec{(x}^{\varvec{\Delta , 7}}\varvec{)}$	(2), l=7
ADAM-iETSX	0.57	7.76	0.83	0.88	0.57	7.49	0.78	0.87
Benchmark-1	0.45	5.05	0.50	0.80	0.44	4.43	0.47	0.78
Benchmark-2	0.51	5.11	0.52	0.80	0.50	4.29	0.45	0.78
ETS	0.58	7.49	0.78	0.87	0.58	7.68	0.84	0.88
GBM-2	0.39	4.93	0.51	0.80	0.37	4.61	0.49	0.79
NBI-2	0.53	5.04	0.52	0.81	0.53	4.41	0.48	0.79
NOtr-1	0.52	5.03	0.51	0.81	0.51	4.41	0.49	0.79
NOtr-2	0.52	5.03	0.51	0.81	0.51	4.41	0.49	0.79
Poisson-1	0.53	5.04	0.51	0.81	0.52	4.38	0.48	0.79
Poisson-2	0.53	5.05	0.52	0.80	0.53	4.42	0.48	0.78
Poisson-2-I	0.51	5.03	0.51	0.81	0.50	4.42	0.49	0.79
Prophet	0.62	5.27	1.00	0.91	0.62	5.15	1.00	0.91
Regression-Poisson	0.51	6.65	0.67	0.85	0.51	6.49	0.67	0.85
Ttr-2	0.51	5.03	0.50	0.81	0.50	4.41	0.49	0.79
qreg-1	0.39	5.01	0.49	0.81	0.39	4.84	0.51	0.80
tbats	0.63	5.35	1.00	0.92	0.63	5.04	1.00	0.92
True	0.54	6.61	1.00	0.93	0.55	5.90	1.00	0.92

The column (1) shows the fraction of values greater than zero for horizon *l*, $\sigma _{x^{\Delta , l}}$ the standard deviation, and $q_{0.1} (x^{\Delta , l})$ the 10% quantile of the changes’ absolute values. Column (2) shows the fraction of values not in the exclusion area of size one

Table 6 lists the ATC ratios for all models for three and seven-day horizons. The ATC ratios range from 0.68 to 0.84 for a horizon of three days and from 0.68 to 0.82 for seven days. The negative and positive ATC ratios differ for all models and horizons. For some models, for example, the GBM-2 model, the positive ATC ratio is higher than the negative ATC ratio, and for some models, for example, the tbats model, vice versa. The confidence interval width is at most 0.02 for the ATC ratios and at most 0.03 for the positive and negative ATC ratios. The models GBM-2, qreg-1, and Benchmark-1 have the highest positive ATC ratio for the three and seven-day horizons, while Poisson-2 and NBI-2 have the highest negative ATC ratio.

**Fig. 5:**
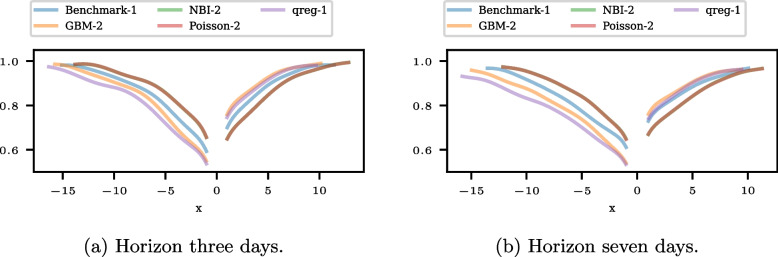
Conditional ATC plots for the horizons three and seven days and the models with the best positive or negative ATC in forecasting the patient admissions to an emergency department. The plots of NBI-2 and Poisson-2 are indistinguishable

Figure 5 shows the conditional ATC plots for the models Benchmark-1, GBM-2, NBI-2, Poisson-2, and qreg-1 for the horizons three and seven days and thus inspects the local ATC of the models with highest positive and negative ATC ratio. The conditional ATC plots show similar courses for the two horizons, though the curves are shifted downwards for the horizon of seven days. The model’s relative ATC evolves consistently for the two horizons, with the NBI-2 and Poisson-2 models being indistinguishable. The GBM-2 model outperforms the qreg-1 model for all predicted changes. The models NBI-2 and Poisson-2 have the highest ATC for all negative predicted changes and the lowest for all positive predicted changes. Benchmark-1 lies between the other models for all predicted changes.

**Fig. 6:**
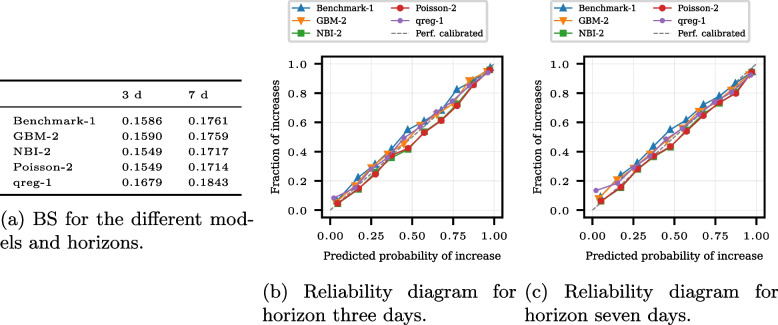
Probabilistic ATC assessment for the models Benchmark-1, GBM-2, NBI-2, Poisson-2, and qreg-1 for the horizons three and seven days in forecasting patient admissions to an emergency department. The Brier score in Fig. 6**a** evaluates the calibration and sharpness of the probabilistic ATC simultaneously, while the two plots on the right assess solely the calibration, that is, whether the predicted probability of increase occurs empirically. Probabilistic ATC by the BS is best for the models NBI-2 and Poisson-2 for both lags

Figure 6 visualizes the probabilistic ATC assessment for the same subset of models. The Brier scores (BSs) are shown in Fig. 6a, and the reliability diagrams for the horizons three and seven days in Fig. 6b and c. The BSs are smallest for NBI-2 and Poisson-2 for both horizons, while the BSs for the other models are larger and differ more. The qreg-1 model has both horizons’ highest BS. The reliability diagrams of GBM-2 and NBI-2 are also close and show a too-small fraction of increases for the predicted probability overall. For the other models, the reliability diagrams show a fraction of increases that are too large for the corresponding predicted probability.

**Table 6:** ATC ratio $\mu _+$, positive ATC ratio $\mu ^{+}_+$, and negative ATC ratio $\mu ^{-}_+$ for the models for the horizons 72 hours and seven days in the forecasting of patient admissions to an emergency department with an exclusion zone equal to the axis indicated by the subscript +

	$\varvec{\mu }^{\varvec{3}}_{\varvec{+}}$	$\varvec{\mu }^{\varvec{+, 3}}_{\varvec{+}}$	$\varvec{\mu }^{\varvec{-, 3}}_{\varvec{+}}$	$\varvec{\mu }^{\varvec{7}}_{\varvec{+}}$	$\varvec{\mu }^{\varvec{+, 7}}_{\varvec{+}}$	$\varvec{\mu }^{\varvec{-, 7}}_{\varvec{+}}$
ADAM-iETSX	0.70 (0.69, 0.71)	0.68 (0.67, 0.69)	0.72 (0.71, 0.73)	0.68 (0.67, 0.69)	0.67 (0.66, 0.69)	0.69 (0.67, 0.70)
Benchmark-1	0.83 (0.82, 0.84)	0.86 (0.85, 0.87)	0.81 (0.79, 0.82)	0.81 (0.80, 0.82)	0.86 (0.85, 0.87)	0.78 (0.76, 0.79)
Benchmark-2	0.84 (0.83, 0.84)	0.83 (0.82, 0.85)	0.84 (0.83, 0.85)	0.82 (0.81, 0.83)	0.83 (0.82, 0.84)	0.80 (0.79, 0.82)
ETS	0.68 (0.67, 0.69)	0.66 (0.65, 0.67)	0.70 (0.69, 0.72)	0.67 (0.66, 0.68)	0.66 (0.64, 0.67)	0.68 (0.66, 0.69)
GBM-2	0.82 (0.81, 0.82)	0.90 (0.89, 0.91)	0.77 (0.76, 0.78)	0.78 (0.77, 0.79)	0.88 (0.87, 0.90)	0.73 (0.72, 0.74)
NBI-2	0.84 (0.83, 0.85)	0.83 (0.82, 0.84)	0.85 (0.84, 0.86)	0.82 (0.81, 0.83)	0.82 (0.81, 0.83)	0.82 (0.81, 0.83)
NOtr-1	0.83 (0.83, 0.84)	0.83 (0.82, 0.84)	0.84 (0.82, 0.85)	0.81 (0.80, 0.82)	0.82 (0.81, 0.83)	0.80 (0.79, 0.81)
NOtr-2	0.83 (0.83, 0.84)	0.83 (0.82, 0.84)	0.84 (0.82, 0.85)	0.81 (0.80, 0.82)	0.82 (0.81, 0.83)	0.80 (0.79, 0.81)
Poisson-1	0.84 (0.83, 0.84)	0.82 (0.81, 0.83)	0.85 (0.84, 0.86)	0.82 (0.81, 0.82)	0.82 (0.81, 0.83)	0.81 (0.80, 0.82)
Poisson-2	0.84 (0.83, 0.85)	0.83 (0.82, 0.84)	0.85 (0.84, 0.86)	0.82 (0.81, 0.82)	0.82 (0.81, 0.83)	0.82 (0.80, 0.83)
Poisson-2-I	0.83 (0.83, 0.84)	0.84 (0.83, 0.85)	0.83 (0.82, 0.84)	0.81 (0.80, 0.82)	0.83 (0.81, 0.84)	0.80 (0.79, 0.81)
Prophet	0.75 (0.74, 0.76)	0.72 (0.71, 0.73)	0.79 (0.77, 0.80)	0.74 (0.73, 0.74)	0.72 (0.70, 0.73)	0.76 (0.75, 0.77)
Regression-Poisson	0.72 (0.71, 0.73)	0.73 (0.71, 0.74)	0.72 (0.70, 0.73)	0.70 (0.69, 0.71)	0.71 (0.70, 0.73)	0.69 (0.67, 0.70)
Ttr-2	0.84 (0.83, 0.84)	0.84 (0.83, 0.85)	0.83 (0.82, 0.85)	0.81 (0.80, 0.82)	0.83 (0.82, 0.84)	0.80 (0.79, 0.81)
qreg-1	0.80 (0.79, 0.80)	0.88 (0.87, 0.89)	0.75 (0.74, 0.76)	0.77 (0.76, 0.78)	0.86 (0.85, 0.88)	0.71 (0.70, 0.72)
tbats	0.75 (0.74, 0.76)	0.72 (0.71, 0.73)	0.80 (0.78, 0.81)	0.73 (0.72, 0.74)	0.71 (0.69, 0.72)	0.76 (0.74, 0.77)

There are 4030 positive changes and 4051 negative changes for the horizon of 72 hours and 4042 and 3961 for the horizon of 7 days

#### Discussion

The ATC is consistent for the two horizons, with the models’ relative ATC evolving similarly for the two horizons. The models’ ATC is generally higher for the smaller horizon, but the changes are minor, and confidence intervals overlap.

The positive and negative ATC ratios differ for all models. While some models, such as GBM-2 and qreg-1, have the highest positive ATC ratio, others, such as Poisson-2 and NBI-2, have the highest negative ATC ratio. Thus, the uncertainty of the model’s predicted change has to be assessed differently based on the direction.

The probabilistic ATC assessment results endorse the point ATC assessment and assign the best scores to NBI-2 and Poisson-2. The reliability diagrams show that they underestimate the fraction of increases slightly.

Overall, the example provides performance assessments that are different from standard point evaluation measures and thus provide further insights into the strengths and weaknesses of the models. While the models with the lowest RMSE, NBI-2, and Poisson-2, also have a high ATC, three models with below-average point evaluation measures, Benchmark-1, GBM-2, and qreg-1, have a high positive ATC.

### Non-invasive blood pressure monitoring

We here consider the ATC of non-invasive blood pressure measurements from the MIMIC-III database that includes data of critically ill patients treated in intensive care units of the Beth Israel Deaconess Medical Center in Boston (Massachusetts, USA, [40, 41]; available through [42]). We focus on arterial blood pressure (ABP) and non-invasive blood pressure (NBP) measurements and thus limit our analysis to datasets containing simultaneous measurements of ABP and NBP simultaneously. 2,548 datasets include at least one measurement of systolic ABP and NBP, and 1,327 include at least one pair of simultaneously measured systolic ABP and NBP; for the mean ABP and NBP, the numbers are 2,605 and 1,516, respectively. We assess the ATC of non-invasive blood pressure measurements (test method) compared to intraarterial blood pressure measurements (reference method, gold standard). We consider the horizons of one minute, five minutes, and 15 minutes for the ATC assessment, as those are typical intervals of NBP measurements.

While ATC assessment is well-established in measurement analysis, our new methods extend its application, revealing insights such as asymmetries. This extension enhances the utility of ATC assessment beyond its conventional use, offering a more comprehensive evaluation of model performance.

#### Results

**Fig. 7:**
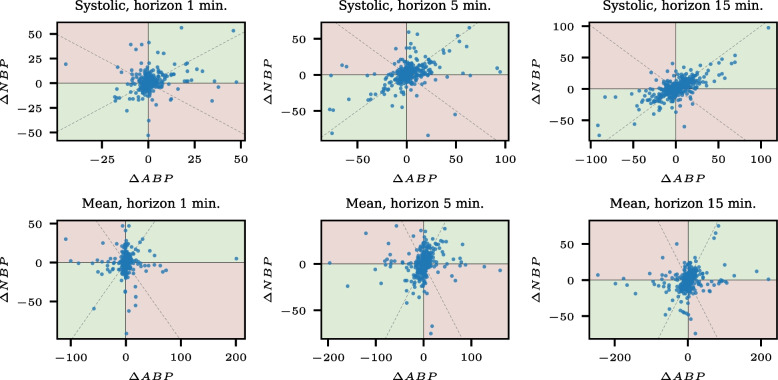
Four-quadrant plots for the different horizons *l* and the systolic and mean blood pressure measurements. The upper row contains systolic measurements, and the lower row contains mean measurements. The columns contain the horizons one, five, and 15 minutes

Again, we exclude the smallest 10% of absolute changes in ATC assessment. The resulting four-quadrant plots of the mean and systolic blood pressure measurements for the different horizons are shown in Fig. 7. The number of points in the four-quadrant plot is smaller due to the restriction to data records with measurements of mean or systolic ABP and NBP simultaneously for two consecutive times with the specified horizons. Thus, we use the NBP measurements as the test method and the ABP measurements as the gold standard. For the systolic measurements, 290, 332, and 442 points are available for the horizons of one, five, and 15 minutes; for the mean measurements, 406, 430, and 542.

The ATC ratios, including confidence intervals for the different horizons, are listed in Table 7. The confidence intervals have lower bounds of 0.5 or slightly above for the measurements with a horizon of one minute. For larger horizons, the ATC ratio increases. The difference between positive and negative ATC ratios is small for all types and horizons, with overlapping confidence intervals.

**Table 7:** ATC ratios for the different horizons *l* and the systolic and mean blood pressure measurements

Type	$\varvec{l}$	$\varvec{\mu }^{\varvec{l}}$	$\varvec{\mu }^{\varvec{+, l}}$	$\varvec{\mu }^{\varvec{-, l}}$
Systolic	1	0.55 (0.50, 0.60)	0.59 (0.52, 0.65)	0.58 (0.50, 0.66)
Systolic	5	0.63 (0.59, 0.68)	0.70 (0.64, 0.75)	0.62 (0.56, 0.69)
Systolic	15	0.69 (0.65, 0.73)	0.72 (0.66, 0.76)	0.74 (0.69, 0.79)
Mean	1	0.55 (0.51, 0.59)	0.62 (0.56, 0.68)	0.56 (0.50, 0.62)
Mean	5	0.59 (0.55, 0.64)	0.65 (0.59, 0.71)	0.62 (0.56, 0.68)
Mean	15	0.62 (0.58, 0.65)	0.65 (0.60, 0.70)	0.66 (0.61, 0.71)

**Fig. 8:**
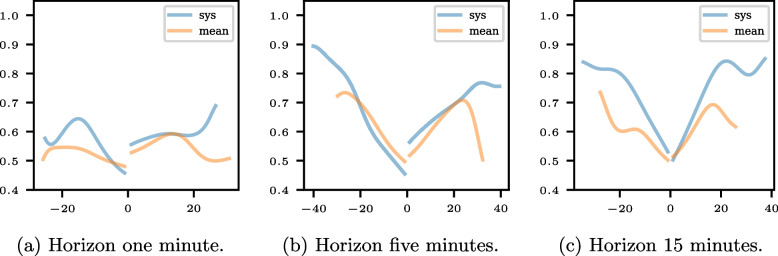
Conditional ATC plot for the systolic and mean blood pressure measurements and the horizons one, five, and 15 minutes

Figure 8 shows the conditional ATC plots for the different horizons and the systolic and mean blood pressure measurements. It becomes apparent that the systolic measurements have a higher ATC than the mean measurements, except for small negative predicted changes.

#### Discussion

The four-quadrant plots contain a considerable number of extreme points. Whether these points are due to measurement errors or extreme values is not distinguishable. Some authors argue to exclude the measurements below the 10%-quantile of the absolute changes and the points above the 90%-quantile (see [20]). We do not follow this approach here, as the extreme values are not necessarily measurement errors and could be particularly relevant.

The difference between positive and negative ATC ratios is small in this example. The positive and negative ATC ratios have overlapping confidence intervals, the conditional ATC plots do not contain prominent deviations in the course, and the four-quadrant plots do not display asymmetries.

The bootstrap confidence intervals are wide. The width is around 0.1 for the ATC ratio, while it gets up to 0.16 for the negative ATC ratio for systolic measurement and the horizon of one minute. Thus, the confidence intervals cover 0.5 for systolic measurement and the horizon of one minute, and the equality to random guessing cannot be excluded.

## Discussion and conclusion

In this paper, we examine various methods to assess the ability to track changes (ATC) for measurements, nowcasts, or forecasts, that is, whether they correctly predict the direction of changes in values. While the computation of predicted change varies between the application areas of measurement, nowcasting, and forecasting, the assessment can be based on the same methods. The ATC assessment can accompany other evaluation techniques, such as measures of deviation or probabilistic scoring rules.

Four-quadrant plots facilitate the visual inspection of the ATC for a measurement, nowcast, or forecast (see The four-quadrant plot section). The ATC ratio, the ratio of change directions predicted correctly over the total number of changes, numerically evaluates ATC. Visually, it is the proportion of concordant points in a four-quadrant plot (see The ATC ratio and other measures section). The positive and negative ATC ratios analyze the ATC ratio given whether the predicted change is positive or negative, respectively. Thus, they quantify the credibility of the respective predictions. The applications of Non-invasive blood pressure monitoring section show that models, in general, indeed have different positive and negative ATC and that they add valuable information to the ATC ratio. In the applications, the bootstrap confidence intervals of The ATC ratio and other measures section are used to quantify the estimation uncertainty of the ATC measures. The width of the confidence intervals is around 0.1 for around 100 samples, while it is around 0.01 for 8000 samples. For models with reasonably high ATC, 100 samples are thus sufficient to differentiate from random guessing or to assess models with high ATC differences.

A conditional ATC plot visualizes the probability of correct change direction prediction over the predicted change of the measurement, nowcast, or forecast (see The conditional ATC plot section). It is based on a multivariate kernel density estimation (KDE) of predicted and observed change. In the application, the conditional ATC plot gives reasonable insights into the local effects of the ATC. Probabilistic evaluation section adapts measures of probabilistic forecast evaluation to the ATC assessment of probabilistic forecasts and nowcasts. The Brier score (BS) as numerical assessment of probabilistic ATC is introduced, and reliability diagrams are used to visualize the local ATC of probabilistic forecasts.

The methods of ATC assessment are applied to COVID-19-nowcasting, forecasting the patient admissions to an emergency department, and invasive and non-invasive blood pressure measurements in Application to medical/healthcare nowcasting, forecasting, and measurement data section. While ATC assessment should not be the only aspect, it is a valuable addition to evaluating nowcasts, forecasts, and measurements. Models with highly different accuracies are usually scored similarly in ATC assessment, but ATC assessment can differentiate between models with similar accuracies. As in the application in Nowcasting during the COVID-19 pandemic section, models with medial point forecast evaluation measures can have the most meaningful positive ATC.

For public health officials and forecasters analyzing methods, we recommend presenting four-quadrant plots, ATC ratios and the conditional ATC plots as a fundamental approach. This presentation makes a model’s ATC easily interpretable and and reveals performance asymmetries. An exclusion area can be incorporated to address for non-relevant changes.

We did not expand on two modeling aspects throughout this paper, which we leave for further research. In the estimation, we did not consider sequential correlation. The computation of differences is a standard procedure in time series analysis to remove sequential dependence, but, in general, some could remain, and the estimators could account for it. Similarly, the bootstrap confidence intervals could be adapted to consider sequential correlation using time-series bootstrap methods [43, 44].

The estimators of The ATC ratio and other measures section do not account for imbalances in the number of observed positive and negative changes (for theoretical analysis, see [28], Chapter 3). Significant differences in the number of observed positive and negative changes are unlikely in the ATC setting, as $\textbf{y}^{\Delta }$ is obtained from differencing time series data and occur, for example, if the true value contains a few high jumps in one direction and many smaller jumps in the other. However, if the number of positive and negative observed changes differs widely, unbalanced-data-aware measures should be considered. There are various adapted measures for unbalanced outcomes, for example, Cohen’s $\kappa$ [45] or those listed in Jolliffe and Stephenson ([28], Table 3.3).

## Appendix A: Additional material on Assessment of the ability to track changes (ATC) section

### Data generation for Assessment of the ability to track changes (ATC) section

The first dataset is generated by sequentially generating $\textbf{x}^{\Delta }$ and $\textbf{y}^{\Delta }$. First, the $x^{\Delta}_{t}$ are sampled as a sum of a standard normal random number and a uniform random number on $(-10, 10)$:

$$\begin{aligned} x^{\Delta}_{t} \sim N(0, 1) + U(-10, 10) \quad t = 1, \dots , T. \end{aligned}$$

Subsequently, the $\textbf{y}^{\Delta }$ are simulated for a constant ATC ratio *k* by

$$\begin{aligned} y^{\Delta}_{t} = x^{\Delta}_{t} \cdot n_t \cdot b_t, \end{aligned}$$

where $n_t$ is a truncated normal distribution with mean one and standard deviation 0.5, truncated at 0, and $b_t$ is a symmetric Bernoulli random variable with parameter *k*. For a time-varying ATC ratio, the parameter *k* is modified to have a wave-shape function over time, that is,

$$\begin{aligned} k_t = 0.75 + \sin (t / 365.25 \cdot 2 \pi ) / 4. \end{aligned}$$

For the asymmetric ATC ratio, *k* is a function of $x^{\Delta}_{t}$,

$$\begin{aligned} k(x) = 0.5 + \min \left\{ \max \left\{ \frac{x + 5}{10}, 0 \right\} , 1 \right\} / 2. \end{aligned}$$

In the second approach, $y^{\Delta}_{t}$ and $x^{\Delta}_{t}$ are modeled to be multivariate normal with mean 0 and covariance matrix

$$\begin{aligned} \Sigma = \left( \begin{array}{cc} 4 & 3 \\ 3 & 4 \end{array}\right) . \end{aligned}$$

Thus, the conditional probability of correct direction prediction can be calculated by a conditional normal distribution to

$$\begin{aligned} P(Y^{\Delta } X^{\Delta }> 0 | X^{\Delta } = x) = \Phi \left( \frac{3}{2 \sqrt{7}} x \right) , \end{aligned}$$

where $\Phi$ is a standard normal CDF.

**Fig. 9:**
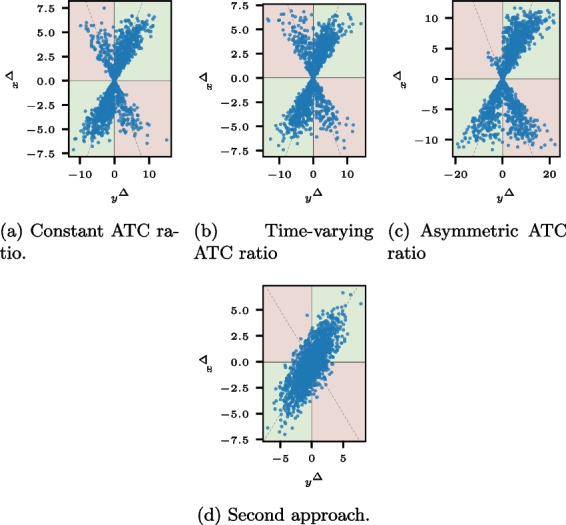
Four-quadrant plots for sample realizations of the data generation schemes of Appendix A: Data generation for Assessment of the ability to track changes (ATC) section. Although the first and second plots differ over time, their difference is not discernible in the plots. The third data set’s asymmetry is visible in the plot, but the decrease in the ATC near 0 is not visible

The four-quadrant plots for the sample realizations of the data generation schemes are shown in Fig. 9.

### Simulation study on bootstrapping confidence intervals

We examine three methods for bootstrapping for computing confidence intervals for the ATC ratio: the intuitive percentile and the more sophisticated basic and BCa method. In the *percentile* approach, the confidence interval for the level $\alpha$ is built directly from the empirical distribution of the bootstrap estimators. The *basic* approach computes the confidence interval based on the non-bootstrap estimate using the bootstrapped quantile deviations [46]. The BCa method modifies the quantiles of the empirical bootstrap distribution by a bias and an acceleration parameter [47]. Typically, the percentile approach requires larger datasets and provides an easy and fast estimate, while the BCa, though computationally expensive, can produce reasonable confidence intervals with smaller datasets. The basic approach balances these two objectives. We compare the approaches in a small synthetic data study on their small-dataset behavior and computation time.

**Fig. 10:**
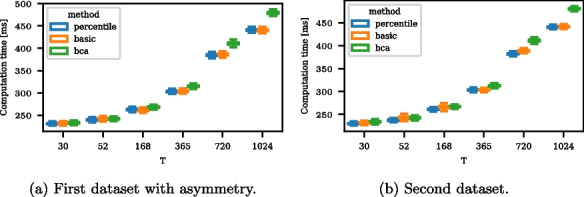
Boxplot of the computation time for different bootstrapping methods and data set sizes *T*. The computation time refers to bootstrapping one confidence interval based upon 10,000 values. Each boxplot reflects 10,000 samples. The BCa method takes slightly longer than the other two, but the difference is negligible

We vary the number of time steps *T* to take typical time-series values, such as 30 for daily data in a month, 52 for weekly data, 168, 365, 720, and 1024. The considered datasets are the first dataset with asymmetric dependence and the second dataset outlined in Appendix A: Data generation for Assessment of the ability to track changes (ATC) section. In the calculations, the |scipy| package’s implementation of bootstrap confidence intervals is used [48]. The prescribed confidence level is 90 %, and the number of bootstrap samples is 10, 000. The share of confidence intervals covering the true values per method and *T* are shown in Table 8. The true values of the accuracy are computed based on a dataset of size $10^8$, yielding 0.7501 and 0.7700 for the two datasets. The computation times per method and dataset are shown in Fig. 10. For the small sample sizes up to $T = 168$, only the BCa method keeps the confidence interval size and yields slightly wider confidence intervals. The method’s results are similar for the larger sample sizes. The computation time for the BCa method is slightly larger than for the other methods, but all methods have a moderate computation time. BCa is the only method that maintains the confidence level for small datasets while increasing the computation time only moderately for larger datasets. Therefore, we use the BCa method for confidence intervals in the applications in Application to medical/healthcare nowcasting, forecasting, and measurement data section.

**Table 8:** Proportion of bootstrap confidence intervals covering the true value of ATC ratio per method and sample size *T*

(a) First dataset.				(b) Second dataset.			
	percentile	basic	BCa		percentile	basic	BCa
30	0.84 (0.249)	0.86 (0.250)	0.91 (0.257)	30	0.87 (0.243)	0.88 (0.242)	0.92 (0.249)
52	0.89 (0.194)	0.89 (0.193)	0.89 (0.198)	52	0.87 (0.188)	0.89 (0.188)	0.90 (0.192)
168	0.91 (0.109)	0.90 (0.109)	0.90 (0.110)	168	0.89 (0.106)	0.90 (0.106)	0.90 (0.107)
365	0.90 (0.074)	0.90 (0.074)	0.90 (0.074)	365	0.90 (0.072)	0.90 (0.072)	0.90 (0.072)
720	0.90 (0.053)	0.90 (0.053)	0.90 (0.053)	720	0.90 (0.052)	0.90 (0.052)	0.90 (0.052)
1024	0.90 (0.044)	0.90 (0.044)	0.89 (0.044)	1024	0.89 (0.043)	0.90 (0.043)	0.90 (0.043)

The average width of the confidence interval is listed in brackets

### Visualization of different bandwidth selectors in multivariate KDE

**Fig. 11:**
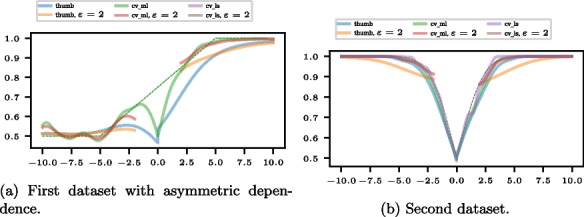
Conditional ATC plot for different bandwidth selection processes. Cross-validation least squares takes a considerably larger computation time. It converges neither for the first nor the second data set with an exclusion area and yields a bandwidth too small for the second data set. The rule of thumb is the fastest method but tends to oversmooth. The cross-validation maximum likelihood method yields a more reasonable bandwidth with moderate computation time. $\varepsilon$ specifies an exclusion area $E = \{(x, y) \in \mathbb {R}^2: (-\varepsilon \leqslant x \leqslant \varepsilon )\}$ in $\textbf{x}^{\Delta }$-direction

**Fig. 12:**
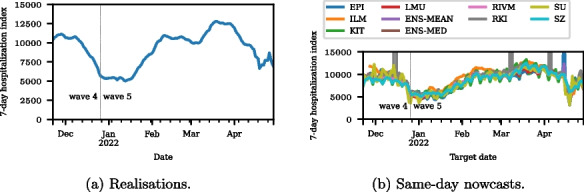
True and nowcast data of the seven-day-hospitalization in Germany from November 22, 2021, to April 29, 2022 [38]. The outliers in the RKI model of values above $10^8$ are removed before the following analysis

**Fig. 14:**
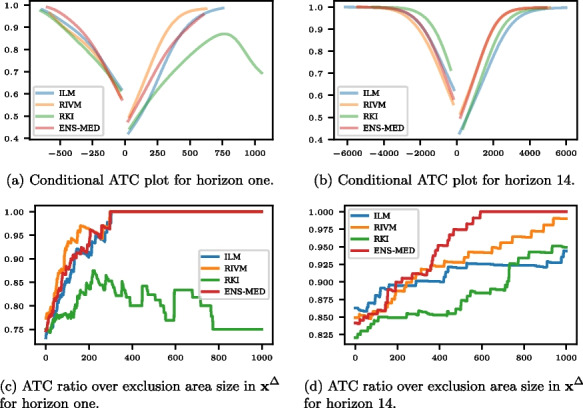
Conditional ATC plot and ATC ratio over exclusion area for the COVID-19 nowcast models ILM, RKI, RIVM, and ENS-MED for the horizon seven days. Note the different axis scalings of the ATC ratios for the two horizons. While the ENS-MED model has a rather low ATC ratio for small exclusion areas, it is increasing fast for larger exclusion areas. The RKI model is among the worst, except for the small negative predicted changes

**Fig. 15:**
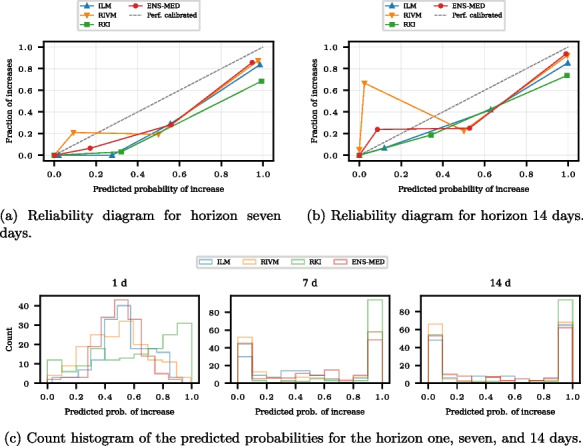
The reliability diagram for the COVID-19 nowcasting models ILM, RIVM, RKI, and ENS-MED for the horizon seven and 14 days. Additionally, the count of predicted probabilities for the horizons is shown. The reliability diagram bins are chosen according to the empirical quantiles of the predicted probabilities. As the models issue small or large probabilities of increase for the higher horizons, little information on the accuracy of moderate probability predictions is available

We examine the resulting conditional ATC plots for the three well-known KDE bandwidth selectors, rule-of-thumb, cross-validation maximum likelihood, and cross-validation least squares using the |statsmodels| Python package [32] in Fig. 11. While the rule-of-thumb is based only on the covariance matrix, the other two numerically optimize the bandwidth with a hold-one-out least squares or likelihood objective function. The dashed line shows the theoretical $P(Y^{\Delta } X^{\Delta }> 0 | X^{\Delta } = \chi )$. The second method, cross-validation least squares, requires long computation times while yielding small or no bandwidth results, even for two relatively small datasets. The rule-of-thumb and cross-validation maximum likelihood methods yield reasonable results at moderate computation times.

### Probabilistic ATC evaluation

Probabilistic evaluation section outlines the assessment of probabilistic ATC for nowcasts and forecasts and specifies the computation for predictions in terms of a CDF and known true values. Here, we outline the computation for quantile forecasts and yet unknown, probabilistic true values.

If forecasts or nowcasts are given as quantiles, $p_t$ can be determined by interpolations among the quantiles. Let $q_p$ denote the quantiles for target time $t+l$ for even-spaced probabilities $p \in \{1/\hat{p}, \dots , (p-1) / \hat{p}\}$ ($\hat{p} \in \mathbb {N} \setminus \{1, 2\}$) and $y_t$ the true value at time *t*. The quantiles $q_p$ generally differ for each time step, but we omit an index here for ease of notation. The probability $p^{c}_t$ of a *negative* change is between $p^{\star }$ and $p^{\star } + 1/\hat{p}$ for

$$\begin{aligned} p^{\star } = \max \{p \in \{1/\hat{p}, \dots , (\hat{p}-1) / \hat{p}\}: q_p \leqslant y_t\} , \quad \text {if}\ q_{1/\hat{p}} \leqslant y_t \leqslant q_{1 - 1/\hat{p}}. \end{aligned}$$

Quantiles do not determine the location within the interval $[p^{\star }, p^{\star } + 1/\hat{p}]$. Under the assumption of a uniform distribution within the quantile interval, the probability of a negative change is

$$\begin{aligned} p^{c}_{t} = \frac{y_t - q_{p^\star }}{\hat{p} (q_{p^{\star } + 1} - q_{p^{\star }})} + p^{\star }. \end{aligned}$$

The approach does not yet assign probabilities for $y_t$ smaller than the smallest quantile $q_{1/p}$ or greater than the largest quantile. As a simple extension, we assume that the probability mass is uniformly distributed on an interval of the same length as the nearest interval specified by the quantiles. This yields

$$\begin{aligned} p^{c}_{t} = \left\{ \begin{array}{ll} \max \{\frac{1}{\hat{p}} - \frac{q_{p^\star } - y_t}{\hat{p} (q_{p2/\hat{p}} - q_{1/\hat{p}})}, 0\} & , \text {if } y_t < q_{1/p}, \\ \min \{\frac{1}{\hat{p}} - \frac{y_t - q_{(\hat{p}-1)/\hat{p}}}{\hat{p} (q_{(\hat{p}-1)/\hat{p}} - q_{(\hat{p}-2)/\hat{p}})}, 1\} & , \text {if } y_t> q_{1 - 1/p}, \\ \frac{y_t - q_{p^\star }}{\hat{p} (q_{p^{\star } + 1} - q_{p^{\star }})} + p^{\star } & , \text {otherwise.} \end{array}\right. \end{aligned}$$

The probability of positive change is $p_t = 1 - p^{c}_{t}$.

If the true value is given as a distribution because it is still unknown, the probabilities $p_t$ can be computed by integration. Let for two nowcasts the distributions be given by PDFs $f_{t+l|t+l}$ and $f_{t|t+l}$ with CDFs $F_{t+l|t+l}$ and $F_{t|t+l}$. Then, the probability of a negative change can be computed by

11 $$\begin{aligned} p^{c}_{t} & = \int _{\begin{array}{l} x_1, x_2 \in \mathbb {R}: \\ x_2 < x_1\end{array}} f_{t|t+l} (x_1) f_{t+l|t+l} (x_2) \ \text {d} \, (x_1, x_2) \nonumber \\ & = \int _{x_1 \in \mathbb {R}} \int _{-\infty }^{x_1} f_{t|t+l} (x_1) f_{t+l|t+l} (x_2) \ \text {d} \, x_2 \ \text {d} \, x_1 \nonumber \\ & = \int _{x_1 \in \mathbb {R}} f_{t|t+l} (x_1) F_{t+l|t+l} (x_1) \ \text {d} \, x_2 \ \text {d} \, x_1 . \end{aligned}$$

Thereby, the distributions are assumed to be independent. If the nowcasts have the form of a multivariate distribution, including the dependence of the two PDFs, $f_{t+l|t+l} (x_2)$ has to be replaced by the PDF conditional on $x_1$. As a Monte Carlo approximation of Eq. (11), the probability can also be calculated by sampling from $f_{t+l|t+l}$ and $f_{t|t+l}$ and calculating the fraction of negative changes. For forecasts, the indexes have to be shifted. If no PDFs are available, they can be estimated from the CDF or quantiles, or the CDF or quantiles can be used to generate samples for the Monte Carlo approximation. This approach is applied in Nowcasting during the COVID-19 pandemic section, as the true values are published with a delay of more than 80 days, and the nowcasts are given as quantiles.

## Figures and Tables

**Fig. 13:**
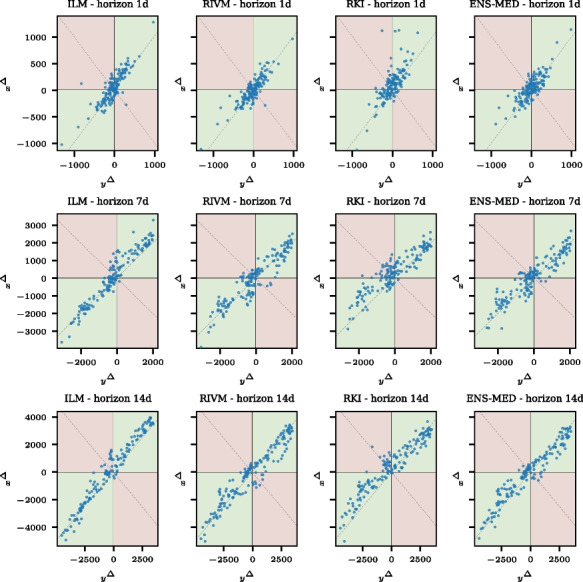
Four-quadrant plots for the COVID-19 nowcast models ILM, RIVM, RKI, and ENS-MEAN and the horizons of one, seven, and 14 days. The spread in both directions increases with the horizon

## Data Availability

The datasets analyzed during the study are available in the corresponding databases or repositories (https://covid19nowcasthub.de/, https://github.com/bahmanrostamitabar/hourly-emergency-care, and https://physionet.org/content/mimic3wdb/1.0/). All code is available in the repository https://github.com/jo-rie/aatc.

## Appendix B: Additional results for Nowcasting during the COVID-19 pandemic section

**Table 9:** Matching the abbreviation to the key in the COVID-19 nowcasting hub

Abbreviation	Nowcasting hub key
EPI	Epiforecasts-independent
ILM	ILM-prop
KIT	KIT-simple_nowcast
LMU	LMU_StaBLab-GAM_nowcast
RIVM	RIVM-KEW
RKI	RKI-weekly_report
SU	SU-hier_bayes
SZ	SZ-hosp_nowcast
ENS-MEAN	NowcastHub-MeanEnsemble
ENS-MED	NowcastHub-MedianEnsemble

Information on the models and references are listed in Wolffram et al. ([10], Table 1)

**Table 10:** Marginal analysis of the COVID-19 nowcast and true changes for the horizons one, seven, and 14 days

	(1), l=1	$\varvec{\sigma }_{\varvec{x}^{\varvec{\Delta , 1}}}$	$\varvec{q}_{\varvec{0.1}} \varvec{(x}^{\varvec{\Delta , 1}}\varvec{)}$	(1), l=7	$\varvec{\sigma }_{\varvec{x}^{\varvec{\Delta , 7}}}$	$\varvec{q}_{\varvec{0.1}} \varvec{(x}^{\varvec{\Delta , 7}}\varvec{)}$	(1), l=14	$\varvec{\sigma }_{\varvec{x}^{\varvec{\Delta , 14}}}$	$\varvec{q}_{\varvec{0.1}} \varvec{(x}^{\varvec{\Delta , 14}})$
EPI	86	520	44	83	1411	78	80	1976	144
ILM	86	281	26	81	1457	102	82	2356	140
KIT	84	354	50	89	1306	171	83	1964	265
LMU	65	285	26	84	1180	124	78	1946	167
ENS-MEAN	85	267	23	86	1213	98	83	1955	235
ENS-MED	88	259	23	88	1206	101	81	1955	186
RIVM	77	241	32	81	1264	123	77	2034	190
RKI	99	362	34	106	1194	145	99	1832	325
SU	91	376	47	85	1390	180	80	2126	263
SZ	92	201	26	89	1154	184	87	1889	241
True	75	262	27	66	1237	126	73	2193	284

The column (1), $l=l$ shows the number of values greater than zero for horizon *l*, $\sigma _{x^{\Delta , l}}$ the standard deviation, and $q_{0.1} (x^{\Delta , l})$ the 10% quantile of the changes’ absolute values

**Table 11:** ATC ratio $\mu$, positive ATC ratio $\mu ^{+}$, and negative ATC ratio $\mu ^{-}$ for the models without and with exclusion areas for the horizon one and 14 days in COVID-19 nowcasting

(a) One day.							(b) 14 days.						
	$\varvec{\mu }^{\varvec{1}}$	$\varvec{\mu }^{\varvec{+, 1}}$	$\varvec{\mu }^{\varvec{-, 1}}$	$\varvec{\mu }^{\varvec{1}}_{\varvec{q}_{\varvec{0.1}}}$	$\varvec{\mu }^{\varvec{+, 1}}_{\varvec{q}_{\varvec{0.1}}}$	$\varvec{\mu }^{\varvec{-, 1}}_{\varvec{q}_{\varvec{0.1}}}$		$\varvec{\mu }^{\varvec{14}}$	$\varvec{\mu }^{\varvec{+, 14}}$	$\varvec{\mu }^{\varvec{-, 14}}$	$\varvec{\mu }^{\varvec{14}}_{\varvec{q}_{\varvec{0.1}}}$	$\varvec{\mu }^{\varvec{+, 14}}_{\varvec{q}_{\varvec{0.1}}}$	$\varvec{\mu }^{\varvec{-, 14}}_{\varvec{q}_{\varvec{0.1}}}$
EPI	0.68 (0.62, 0.74)	0.64 (0.55, 0.72)	0.73 (0.63, 0.81)	0.69 (0.63, 0.75)	0.64 (0.56, 0.73)	0.75 (0.65, 0.82)	EPI	0.83 (0.77, 0.87)	0.79 (0.70, 0.85)	0.87 (0.80, 0.92)	0.85 (0.80, 0.90)	0.81 (0.73, 0.87)	0.90 (0.83, 0.95)
ILM	0.73 (0.67, 0.79)	0.67 (0.58, 0.76)	0.82 (0.73, 0.89)	0.74 (0.68, 0.79)	0.68 (0.60, 0.76)	0.82 (0.72, 0.89)	ILM	0.86 (0.81, 0.90)	0.78 (0.70, 0.85)	0.96 (0.90, 0.99)	0.87 (0.82, 0.91)	0.80 (0.71, 0.86)	0.96 (0.90, 0.99)
KIT	0.62 (0.55, 0.68)	0.58 (0.49, 0.67)	0.65 (0.56, 0.75)	0.62 (0.56, 0.69)	0.59 (0.51, 0.67)	0.66 (0.57, 0.74)	KIT	0.81 (0.75, 0.86)	0.76 (0.67, 0.83)	0.87 (0.79, 0.92)	0.82 (0.76, 0.86)	0.76 (0.68, 0.84)	0.88 (0.81, 0.93)
LMU	0.66 (0.60, 0.72)	0.66 (0.57, 0.75)	0.66 (0.57, 0.73)	0.66 (0.59, 0.72)	0.66 (0.55, 0.75)	0.66 (0.57, 0.73)	LMU	0.88 (0.83, 0.92)	0.85 (0.77, 0.91)	0.91 (0.85, 0.95)	0.89 (0.85, 0.93)	0.87 (0.79, 0.92)	0.91 (0.85, 0.95)
ENS-MEAN	0.81 (0.75, 0.85)	0.76 (0.68, 0.84)	0.88 (0.81, 0.93)	0.81 (0.75, 0.86)	0.76 (0.68, 0.83)	0.88 (0.81, 0.94)	ENS-MEAN	0.83 (0.77, 0.87)	0.77 (0.69, 0.84)	0.89 (0.83, 0.95)	0.84 (0.78, 0.88)	0.78 (0.70, 0.85)	0.91 (0.84, 0.95)
ENS-MED	0.75 (0.68, 0.80)	0.69 (0.60, 0.77)	0.81 (0.73, 0.89)	0.75 (0.69, 0.80)	0.69 (0.61, 0.77)	0.83 (0.74, 0.90)	ENS-MED	0.84 (0.79, 0.89)	0.79 (0.70, 0.85)	0.90 (0.83, 0.95)	0.85 (0.80, 0.90)	0.80 (0.72, 0.86)	0.91 (0.84, 0.96)
RIVM	0.77 (0.72, 0.82)	0.75 (0.66, 0.83)	0.79 (0.71, 0.85)	0.78 (0.72, 0.83)	0.75 (0.66, 0.83)	0.81 (0.73, 0.87)	RIVM	0.85 (0.80, 0.89)	0.82 (0.74, 0.88)	0.88 (0.80, 0.93)	0.85 (0.80, 0.90)	0.83 (0.75, 0.89)	0.88 (0.80, 0.93)
RKI	0.74 (0.68, 0.80)	0.67 (0.59, 0.75)	0.88 (0.79, 0.93)	0.74 (0.67, 0.79)	0.66 (0.58, 0.73)	0.87 (0.78, 0.93)	RKI	0.81 (0.75, 0.86)	0.71 (0.63, 0.77)	0.98 (0.93, 1.00)	0.81 (0.75, 0.86)	0.71 (0.63, 0.78)	1.00 (nan, nan)
SU	0.71 (0.65, 0.77)	0.66 (0.57, 0.74)	0.78 (0.69, 0.85)	0.72 (0.66, 0.78)	0.67 (0.58, 0.75)	0.79 (0.70, 0.87)	SU	0.88 (0.83, 0.92)	0.84 (0.76, 0.90)	0.92 (0.86, 0.96)	0.89 (0.84, 0.93)	0.85 (0.77, 0.91)	0.94 (0.87, 0.97)
SZ	0.74 (0.69, 0.80)	0.68 (0.60, 0.76)	0.82 (0.73, 0.88)	0.74 (0.69, 0.80)	0.68 (0.60, 0.76)	0.82 (0.73, 0.88)	SZ	0.82 (0.77, 0.87)	0.76 (0.68, 0.83)	0.90 (0.83, 0.94)	0.83 (0.78, 0.88)	0.78 (0.69, 0.85)	0.90 (0.83, 0.94)

The exclusion areas are rectangles centered on the zero points with a width and height to exclude the 10%-quantile of the absolute values of nowcast or true values. There are 75 positive and 84 negative actual changes in the considered time period for horizon one day and 73 and 86 for the horizon of 14 days
